# Association of Lipid Levels With COVID-19 Infection, Disease Severity and Mortality: A Systematic Review and Meta-Analysis

**DOI:** 10.3389/fcvm.2022.862999

**Published:** 2022-03-24

**Authors:** Vignesh Chidambaram, Harinivaas Shanmugavel Geetha, Amudha Kumar, Marie Gilbert Majella, Ranjith Kumar Sivakumar, Dinesh Voruganti, Jawahar L. Mehta, Petros C. Karakousis

**Affiliations:** ^1^Department of Medicine, Division of Infectious Diseases, Johns Hopkins School of Medicine, Baltimore, MD, United States; ^2^Department of Internal Medicine, University of Arkansas for Medical Sciences, Little Rock, AR, United States; ^3^Department of Internal Medicine, Saint Vincent Hospital, Worcester, MA, United States; ^4^Department of Community Medicine, Sri Venkateshwaraa Medical College Hospital and Research Centre, Pondicherry, India; ^5^Department of Anaesthesia and Intensive Care, Prince of Wales Hospital, The Chinese University of Hong Kong, Hong Kong, Hong Kong SAR, China; ^6^Division of Cardiovascular Medicine, University of Arkansas for Medical Sciences, Little Rock, AR, United States; ^7^Division of Cardiovascular Medicine, Central Arkansas Veterans Healthcare System, Little Rock, AR, United States; ^8^Department of International Health, Johns Hopkins Bloomberg School of Public Health, Baltimore, MD, United States

**Keywords:** HDL cholesterol, LDL cholesterol, meta-regression, COVID-19, severity, mortality

## Abstract

**Background:**

Coronavirus disease 2019 (COVID-19) ranges from asymptomatic infection to severe illness. Cholesterol in the host cell plasma membrane plays an important role in the SARS-CoV-2 virus entry into cells. Serum lipids, especially low-density lipoprotein cholesterol (LDL-C) and high-density lipoprotein cholesterol (HDL-C), are in constant interaction with the lipid rafts in the host cell membranes and can modify the interaction of virus with host cells and the resultant disease severity. Recent studies on serum lipid levels and COVID-19 disease severity lack consistency.

**Objectives:**

Our systematic review and meta-analysis compared the serum levels of total cholesterol (TC), LDL-C, HDL-C, and triglycerides (TG) between (1) COVID-19 patients vs. healthy controls; (2) severe vs. non-severe COVID-19 disease; (3) deceased vs. surviving COVID-19 patients.

**Methods:**

PRISMA guidelines were followed. We included peer-reviewed articles on observational (case-control and cohort) studies from PubMed and Embase published from the database inception until September 1, 2021. We used random-effects meta-analysis for pooled mean-differences (pMD) in lipid levels (mg/dL) for the above groups.

**Results:**

Among 441 articles identified, 29 articles (26 retrospective and 3 prospective cohorts), with an aggregate of 256,721 participants, were included. COVID-19 patients had lower TC (pMD-14.9, 95%CI-21.6 to −8.3) and HDL-C (pMD-6.9, 95%CI −10.2 to −3.7) levels (mg/dL). Severe COVID-19 patients had lower TC (pMD-10.4, 95%CI −18.7 to −2.2), LDL-C (pMD-4.4, 95%CI −8.4 to −0.42), and HDL-C (pMD-4.4, 95%CI −6.9 to −1.8) at admission compared to patients with non-severe disease. Deceased patients had lower TC (pMD-14.9, 95%CI −21.6 to −8.3), LDL-C (pMD-10.6, 95%CI −16.5 to −4.6) and HDL-C (pMD-2.5, 95%CI −3.9 to −1.0) at admission. TG levels did not differ based on COVID-19 severity or mortality. No publication bias was noted.

**Conclusion:**

We demonstrated lower lipid levels in patients with COVID-19 infection and an association with disease severity and mortality. Their potential role in COVID-19 pathogenesis and their utility as prognostic factors require further investigation.

## Introduction

Coronavirus disease 2019 (COVID-19), caused by SARS-CoV-2, is currently the leading cause of death due to a single infectious agent (1). While the majority of patients infected with COVID-19 are in the asymptomatic or mild category, a considerable proportion develop severe illness, presenting with acute respiratory distress syndrome, renal or hepatic dysfunction, requiring respiratory and multiorgan support in critical care settings (2, 3). Cholesterol is an important component of the membranes of host cells and enveloped viruses and has been shown to play an important role in the entry of SARS-CoV-2 virus into cells (4). Serum lipids, especially low-density lipoprotein cholesterol (LDL-C) and high-density lipoprotein cholesterol (HDL-C). are in constant interaction with the lipid rafts in the host cell membranes (5, 6) and can modify the interaction of virus with host cells and the resultant disease severity.

Several studies have reported an association between COVID-19 severity and low serum levels of HDL-C (7, 8), but the magnitudes of the association between COVID-19 and total cholesterol (TC), LDL-C, and triglycerides (TG) are inconsistent. This finding is in agreement with similar observations in patients with other infections, such as HIV, dengue, and tuberculosis (9, 10). The precise mechanism underlying the relationship between COVID-19 infection and low serum lipid levels is unclear, but the low serum lipid levels have been hypothesized to reflect an acute phase response during inflammation (11). Increased secretory phospholipase and serum amyloid A (SAA), and decreased reverse cholesterol transport have been attributed to low HDL-C levels following inflammation (12–14).

Multiple cardiovascular and metabolic risk factors have been shown to be associated with the risk of severe COVID-19 disease (2). We hypothesized that serum lipid levels may serve as biomarkers predicting disease severity and mortality and provide insight into the pathogenesis of COVID-19. We performed a systematic review of the literature and meta-analysis to determine differences in serum lipid levels, such as TC, LDL-C, HDL-C, and TG, between patients with COVID-19 and healthy controls, and to assess the association of these lipid levels at admission with severity and mortality among patients with COVID-19.

## Methods

### Search Methods and Study Selection

The systematic review was performed following the PRISMA guidelines (15). A literature search was performed in the PubMed and Embase databases on April 20, 2021, and the last literature update was performed on September 1, 2021. The complete search strategy is described in the supplementary document (Appendix Section I). Only articles in the English language were included in this review. Observational studies, such as prospective and retrospective cohort studies, and case-control studies, were included. Authors VC and MM reviewed the study designs of the selected studies. We excluded cross-sectional studies, case reports, and case series from this review. No clinical trial data on COVID-19 were relevant to our research question.

We included studies that reported serum levels of lipids, such as TC, LDL-C, HDL-C, and TG, and at least one of the following: (1) differences in the lipid levels of patients with and without COVID-19; (2) direct comparison of lipid levels at admission among COVID-19 patients with and without severe disease (16, 17); or (3) direct comparison of lipid levels at admission among patients with COVID-19 who died and those who survived.

### Literature Screening

We used the COVIDENCE platform for the systematic review (18). After removing the duplicates, title and abstract screening was done by VC and AK and conflicts were resolved by MM. Full texts of the selected articles were independently screened by two authors (VC, AK) and conflicts were settled by MM. Two of the authors (VC, HN, RK) were independently involved in data extraction and quality assessment and conflicts were resolved by MM. Qualtrics platform was utilized for data extraction (19).

Data were collected on the study characteristics, source of funding, type of lipid assessed (TC, LDL-C, HDL-C, and TG), year of publication, number of centers in each study, and study design. The primary outcomes were the pooled mean differences in lipid levels in the following groups: (a) subjects with and without COVID-19 infection; (b) patients with COVID-19 with and without severe disease; and (c) patients with COVID-19 who died and those who survived. Continuous data for the lipid levels were recorded as means. The median values for the lipid levels available from the studies were transformed into means (20). No secondary outcomes were considered for our systematic review.

Risk assessment for bias was performed independently by two of the authors (VC, MM or RK) using the Newcastle-Ottawa quality assessment scale (NOS) for observational studies (21). Conflicts related to the bias assessment were settled by AK.

### Data Synthesis and Analysis

We obtained pooled mean differences for the groups of interest using random-effects meta-analysis. We analyzed separately the mean levels of TC, LDL-C, HDL-C, and TG for each of the above comparisons. Statistical heterogeneity across the studies was evaluated by forest plots, I^2^ and Tau^2^ statistics. We assessed publication bias using funnel plot and Egger’s test. Meta regression analyses for the mean difference of lipid levels across comparison groups were performed against the mean difference in age or difference in proportions of the confounding parameters. Such as sex, diabetes, hypertension, and coronary artery disease across the groups. All analyses were carried out using the meta package in Stata (StataCorp, version 16) (22).

## Results

### Study Identification and Quality Assessment

**FIGURE 1:**
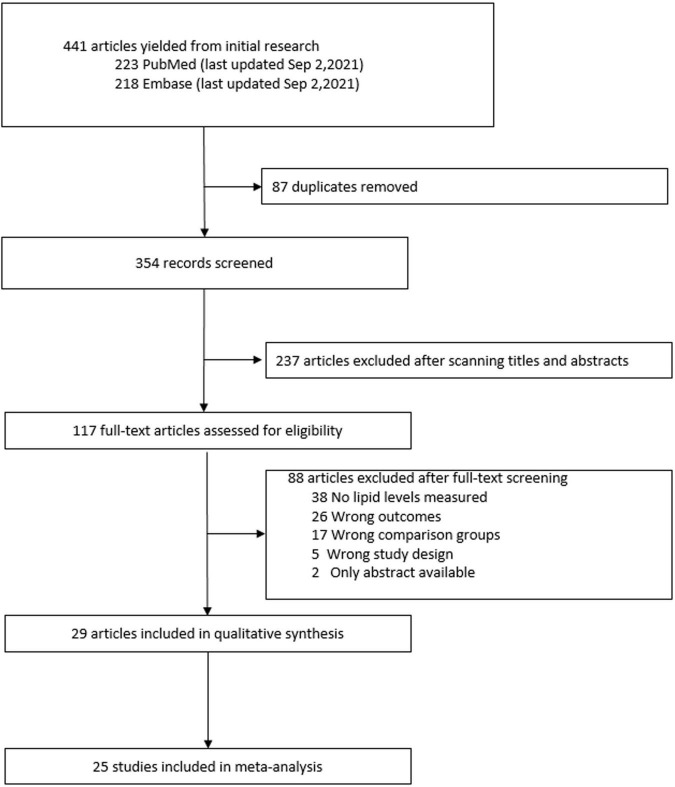
Study selection for the systematic review.

Among the 441 studies obtained from the PubMed and EMBASE databases. After removal of duplicates, we retrieved 354 articles. We assessed full texts of 117 studies and 29 studies met the inclusion criteria. The reasons for exclusion of studies are outlined in Figure 1. Among them, 7 studies compared lipid levels among patients with and without COVID-19, 18 studies reported lipid levels of patients with and without severe disease, and 9 studies reported lipid levels of patients who died compared to those who survived. Of the 29 studies included in the review, 26 were retrospective and 3 were prospective cohorts. In this systematic review, 23 reported TC levels, 26 reported LDL-C levels, 25 studies reported HDL-C levels, and 20 studies reported TG levels. Four articles were not included in the quantitative analysis, as they did not report quantitative differences in the lipid levels for the study outcomes of interest.

Of the studies that assessed lipid levels among patients with or without COVID-19, a total of 2,56,721 participants were available. There were 2,356 patients in studies comparing lipid levels in patients with vs. those without severe COVID-19 disease and 2,127 patients in studies comparing lipid levels in patients who died vs. those who survived. There were 17 studies from China, 3 from France, 2 from Spain, 2 from United Kingdom, and 1 each from United States, Mexico, Italy, Iran, and Turkey. The characteristics of the included studies are listed in Supplementary Table 1 (Appendix Section II). Quality assessment performed using the New-Castle Ottawa scale (NOS) revealed that one study (3.4%) scored 9, 17 studies (58.6%) scored 8, six studies (20.6%) scored 7, and five studies (17.2%) scored 6 or less (Supplementary Table 2—Appendix section II).

**FIGURE 2:**
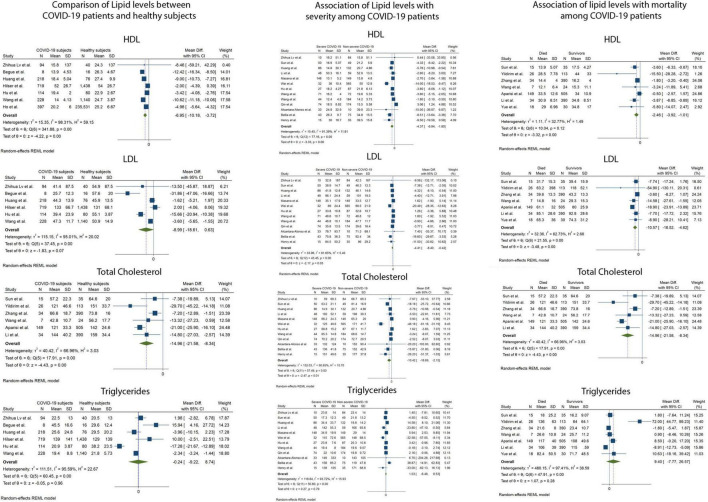
Forest plots showing the random effects meta-analysis for the association of lipid levels with COVID-19 infection, disease severity and mortality. LDL-C, Low density Lipoprotein, HDL-C, High Density Lipoprotein.

Figure 2 and Table 1 depict the pooled mean difference (pMD) of lipid parameters between different comparison groups in the study.

**TABLE 1:** Association of lipid levels with COVID-19 infection, disease severity and mortality.

Serum lipids	Pooled mean difference in lipid levels (mg/dL)				
	No. of studies	No. of patients	Pooled MD [95% CI]	Heterogeneity	
				I^2^	T^2^
**COVID-19 patients vs. Healthy subjects**					
TC	6	4704	−14.9 [−21.6 to −8.3]	66.9%	40.4
LDL-C	6	5679	−8.9 [−18.6 to 0.6]	95.0%	115.2
HDL-C	7	241607	−6.9 [−10.2 to −3.7]	98.3%	15.4
TG	6	5815	−0.2 [−9.2 to 8.7]	95.6%	111.5
**COVID-19 patients: Severe vs. Non-severe**					
TC	12	2213	−10.4 [−18.7 to −2.2]	90.7%	152.0
LDL-C	13	2356	−4.4 [−8.4 to −0.4]	81.7%	33.4
HDL-C	13	2356	−4.4 [−6.9 to −1.8]	91.4%	15.5
TG	12	2213	1.0 [−6.5 to 8.5]	93.7%	118.8
**COVID-19 patients: Died vs. Alive**					
TC	6	2079	−14.9 [−21.6 to −8.3]	66.9%	40.4
LDL-C	7	2127	−10.6 [−16.5 to −4.6]	62.7%	32.4
HDL-C	7	2127	−2.5 [−3.9 to −1.0]	32.8%	1.1
TG	7	2127	9.4 [−7.7 to 26.6]	97.4%	480.2

*CI, Confidence interval; HDL-C, High density lipoproteins; LDL-C, Low density lipoproteins; TC, Total Cholesterol; TG, Triglycerides; MD, Mean difference.*

### Total Cholesterol

Serum TC was reported in 23 of 29 included studies. The pMD in TC levels between COVID-19 patients and healthy controls was −14.9 mg/dL (95% Confidence Interval (CI), −21.6 to −8.3) based on 6 studies (I^2^ = 66.9). Patients with severe COVID-19 disease had lower mean TC levels at admission compared to patients with non-severe disease [pMD −10.4 mg/dL (95%CI −18.7 to −2.2)] based on 12 studies (I^2^ = 90.7%). Mean TC levels at admission in patients who died were lower than those of patients who survived [pMD −14.9 mg/dL (95%CI −21.6 to −8.3)] based on 6 studies (I^2^ = 66.9%).

### Low-Density Lipoprotein Cholesterol

Serum LDL-C was reported in 26 of 29 included studies. The pMD in LDL-C levels between COVID-19 patients and healthy controls was −8.9 mg/dL (95%CI, −18.6 to 0.6) based on 6 studies (I^2^ = 95.0%). Patients with severe COVID-19 disease had lower mean LDL-C levels at admission compared to patients with non-severe disease [pMD −4.4 mg/dL (95%CI −8.4 to −0.42)] based on 13 studies (I^2^ = 81.7%). Mean LDL-C levels at admission in patients who died were lower than those of patients who survived [pMD −10.6 mg/dL (95%CI −16.5 to −4.6)] based on 7 studies (I^2^ = 62.7%).

### High-Density Lipoprotein Cholesterol

Serum HDL-C was reported in 25 of 29 included studies. The pMD in HDL-C levels between COVID-19 patients and healthy controls was −6.9 mg/dL (95%CI, −10.2 to −3.7) based on 7 studies (I^2^ = 98.3%). Patients with severe COVID-19 disease had lower mean HDL-C levels at admission compared to patients with non-severe disease [pMD −4.4 mg/dL (95%CI −6.9 to −1.8)] based on 13 studies (I^2^ = 91.4%). Mean HDL-C levels at admission in patients who died were lower than those of patients who survived [pMD −2.5 mg/dL (95%CI −3.9 to −1.0)] based on 7 studies (I^2^ = 32.8%).

### Triglycerides

**FIGURE 3:**
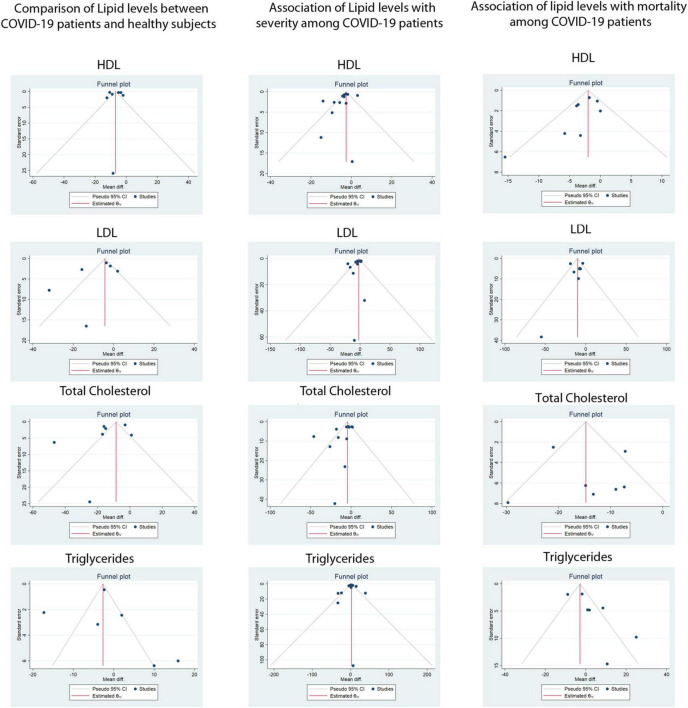
Funnel plots for assessment of publication bias for the association of lipid levels with COVID-19 infection, disease severity and mortality. LDL-C, Low density Lipoprotein, HDL-C, High Density Lipoprotein.

Serum TG was reported in 20 of 29 included studies. The pMD in TG levels between COVID-19 patients and healthy controls was −0.2 mg/dL (95%CI, −9.2 to 8.4) based on 6 studies (I^2^ = 95.6%). Patients with severe COVID-19 disease had similar mean TG levels at admission compared to patients with non-severe disease [pMD −1.0 mg/dL (95%CI –6.5 to 8.5)] based on 12 studies (I^2^ = 93.7%). Mean TG levels at admission in patients who died were similar to those of patients who survived [pMD 9.4 mg/dL (95%CI −7.7 to 26.6)] based on 7 studies (I^2^ = 97.4%). None of the above analyses were found to have significant publication bias using Egger’s test and visual inspection of the funnel plots (Figure 3).

Meta-regression analysis for COVID-19 disease severity did not result in a statistically significant association between the mean difference of either TC, LDL-C, HDL-C, or TG, and the mean difference in age, or difference in proportions of male sex, diabetes, hypertension, or coronary artery disease (Table 2). The bubble plots for the meta-regression analysis are shown in Supplementary Figures 1–5 (Appendix section III). Meta-regression analyses were not performed for the comparison between COVID-19 patients and healthy individuals, and the comparison between COVID-19 patients who died and survived, because of the small number of studies reporting the confounding parameters for these comparisons. The PRISMA checklist for our systematic review is available in Supplementary appendix section IV.

**TABLE 2:** Meta-regression analysis to assess the impact of the confounding variables on the mean difference of lipid levels (mg/dL) between patients with and without severe COVID-19 disease.

Type of lipid	Confounding variable	Number of studies	Meta-regression co-efficient [95%CI]	*p*-value
TC	Age (years) ^#^	11	−0.26 [−1.51, 0.99]	0.682
	Male sex (%) *	10	0.23 [−0.87, 1.33]	0.675
	DM (%) *	6	−0.05 [−1.29, 1.19]	0.934
	HTN (%) *	6	−0.68 [−2.46, 1.10]	0.454
	CAD (%) *	6	−0.74 [−2.61, 1.12]	0.434
LDL-C	Age (years) ^#^	12	0.07 [−0.51, 0.64]	0.824
	Male sex (%) *	11	0.36 [−0.12, 0.85]	0.139
	DM (%) *	7	−0.09 [−0.68, 0.49]	0.749
	HTN (%) *	7	0.20 [−0.36, 0.76]	0.483
	CAD (%) *	7	−0.37 [−1.29, 0.55]	0.431
HDL-C	Age (years) ^#^	12	0.01 [−0.36, 0.36]	0.995
	Male sex (%) *	11	0.01 [−0.31, 0.32]	0.986
	DM (%) *	7	0.03 [−0.34, 0.41]	0.857
	HTN (%) *	7	0.05 [−0.31, 0.41]	0.799
	CAD (%) *	7	−0.14 [−0.75, 0.47]	0.650
TG	Age (years) ^#^	11	0.20 [−1.02, 1.41]	0.750
	Male sex (%) *	10	0.50 [−0.69, 1.48]	0.654
	DM (%) *	6	0.59 [−0.98, 2.16]	0.463
	HTN (%) *	6	−1.86 [−3.60, 0.13]	0.055
	CAD (%) *	6	0.44 [−2.24, 3.12]	0.747

*^#^Mean difference of the variable between severe and non-severe COVID-19 disease.*

*Difference in proportion of the variable between severe and non-severe COVID-19 disease.

*Each covariate fitted into a bivariable meta-regression model.*

*CI, Confidence interval; HDL-C, High density lipoproteins; LDL-C, Low density lipoproteins; TC, Total Cholesterol; TG, Triglycerides.*

## Discussion

In the current study, we found that patients with COVID-19 had lower mean serum TC and HDL-C levels at the time of testing compared to healthy controls (Figure 2 and Table 1). There was no difference in mean LDL-C and TG levels among all patients with and without COVID-19. Lower serum TC, LDL-C, and HDL-C levels at admission were observed in patients with severe COVID-19, as well as in those who died relative to the respective comparison groups. Serum TG levels showed no relationship with COVID-19 severity or mortality. We did not find statistically significant publication bias for any of the analyses.

Lipids play a significant role in respiratory infections. The composition of pulmonary surfactant, which is an integral part of the innate and adaptive immune systems (23, 24), is 90% lipids (25). Moreover, more than 80% of total lung cholesterol is derived from the plasma (26). Of note, lower HDL-C was associated with COVID-19 infection, severity, and mortality in our meta-analysis. HDL-C plays a significant role in host resistance to bacterial, viral and parasitic infections (27, 28) and exhibits multiple properties, such as anti-inflammatory, anti-thrombotic, and anti-oxidative activities (28, 29). HDL-C and its structural protein, apoA-1, have been shown in pre-clinical studies to directly exert anti-inflammatory effects (30, 31) by neutralizing lipopolysaccharide and lipoteichoic acid, and preventing activation of peripheral blood monocytes, thereby decreasing TNF-α and IL-1β synthesis and secretion (32–34).

Oster et al. reported one of the first associations of hypocholesterolemia with increased in-hospital mortality (35). Multiple recent studies have found that patients with various infections, such as cytomegalovirus (36), Epstein-Barr virus (37), and tuberculosis (38), had lower levels of serum lipoprotein compared to non-infected controls. Several studies have established that plasma lipid levels are inversely associated with infection-related and all-cause mortality (10, 39), and have defined the role of lipid levels in acute infectious processes (40). In line with these reports, our meta-analysis highlights the association between COVID-19 infection and lower TC, LDL-C and HDL-C levels.

Recent systematic reviews have also noted findings similar to ours (41, 42), but they have not compared the lipid levels between infected patients and uninfected controls. Additionally, these reviews (41, 42) have only included studies up to January, 2021 while our study included articles up to September, 2021. Meta-regression analysis for the cofounder variables was not performed in the review by Mahat et al. (41), and thus did not account adequately for these variables. In the review by Zinellu et al. (42), the outcomes, namely severe disease and mortality, were combined into a composite outcome, thus the control group for the patients who survived may also contain patients with severe disease leading to a higher degree of heterogeneity in the analysis. Consistent with our findings, the meta-analysis by Ulloque-Badaracco et al. (43) found that serum levels of apoproteins Apo-A1 and Apo-B, which are major apoproteins of HDL-C and LDL-C, respectively, were lower in patients with severe COVID-19.

**FIGURE 4:**
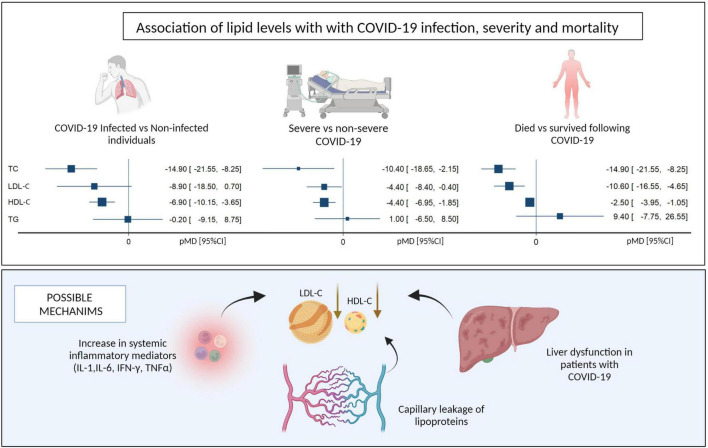
Summary of pooled mean differences and possible mechanisms for the association of lipid levels with COVID-19 infection, disease severity and mortality. LDL-C, Low density Lipoprotein, HDL-C, High Density Lipoprotein (Created with BioRender.com).

Several potential mechanisms explaining the altered serum lipid levels observed during COVID-19 infection have been postulated (Figure 4). Firstly, increased secretory phospholipase and SAA, together with decreased reverse cholesterol transport, have been attributed to low HDL-C levels following inflammation (12–14, 23). SAA-enriched HDL-C is known to be cleared more rapidly from circulation than regular HDL-C through scavenging mechanisms (23). Secondly, the liver plays a critical role in lipid metabolism, and liver dysfunction occurs frequently (in up to 53% cases) in severely ill patients with COVID-19 (44). Furthermore, severe COVID-19 is associated with excessive release of pro-inflammatory cytokines, such as IL-1, IL-6, IFN-γ, and TNF-α, which were shown to decrease the synthesis and secretion of apolipoproteins in hepatic cell lines in a dose-dependent manner (11, 28, 45). Thirdly, severe inflammation can cause leakage of lipoproteins and apolipoproteins in the capillaries (46). Lower serum levels of HDL-C result in a decrease in the anti-inflammatory activities of this molecule, leading to cytokine overproduction and further reduction of HDL-C levels (23). The net effect of this vicious cycle is a more profound depletion of serum HDL-C in the setting of inflammation (8, 34, 47). *In vitro* studies have reported that SARS-CoV-2 can bind to HDL particles *via* the spike protein of the virus (48), but whether this is an important mechanism for lowering HDL cholesterol that is specific to COVID-19 is unclear. Additionally, there are no currently available studies directly comparing the effect of COVID-19 and non-COVID-19 acute respiratory illness on serum lipid levels.

Although LDL-C levels appear to correlate with disease activity in other infections, such as dengue (49) and tuberculosis (10), a similar consistent correlation has not been observed with COVID-19 (50). The studies reporting low LDL-C levels attribute this finding to COVID-19-induced liver injury (44), and increased uptake by monocyte-derived macrophages in patients with COVID-19 (51). We observed a significant association between low serum LDL-C levels and severe COVID-19 infection and mortality. A systematic review noted that patients with dyslipidemia or hyperlipidemia were associated with higher risk of COVID-19 severity and mortality, but the study did not report the precise numerical values of plasma lipids (52).

Although some studies have reported an association between elevated serum TG levels and mortality in COVID-19 (53, 54), our meta-analysis showed that TG levels were not associated with COVID-19 infection, or disease severity or mortality. Of note, TG and its breakdown products, namely fatty acids, activate the NF-κB pathway, with increased expression of pro-inflammatory cytokines, including TNFα, IL-1β, IL-6, and MCP-1 (monocyte chemoattractant protein-1) (55). Also, TG-rich particles in the serum can increase local inflammation, activate the complement pathway and promote endothelial dysfunction (56, 57).

Our study has several strengths. This is the first systematic review to examine the association between lipid levels and the presence, severity, and mortality in COVID-19 infections and assess the robustness of our conclusions through meta-regression for common confounding variables, such as age, sex, diabetes, hypertension, and coronary artery disease. Our systematic review of studies from nine countries with variable healthcare access, allow for generalizability of our results. Our study also has some limitations. First, the information on COVID-19 is highly dynamic, and this is a summary of the currently published articles. Although a pooled analysis of adjusted estimates is desirable, most studies do not give uniform estimates adjusted for similar parameters. Time points of assessment of lipid levels were taken at admission to maintain uniformity but the lipid levels were observed to be dynamic during the time of hospital admission in multiple studies. The included studies did not clearly report whether these lipid levels were taken in the fasting or non-fasting state. The use of lipid modifying agents by the patients in the cohorts were not recorded well in most included studies. Lasty, very few of the studies that assessed changes in serum lipid levels presented data on inflammation and liver injury markers.

Our analysis demonstrated lower lipid levels in patients with COVID-19 compared to healthy controls. Among COVID-19 patients, lower TC, LDL-C and HDL-C levels at admission were associated with disease severity and mortality. Our results might spur further interest in probing the association and interaction of lipid levels and inflammation in atherosclerotic cardiovascular diseases. Current evidence suggests that reduced lipoprotein levels are secondary to systemic inflammation and hepatic dysfunction, but the causal relationship between circulating lipid levels and COVID-19 disease requires further study. Finally, whether low lipid levels prior to hospital admission contribute directly to increased COVID-19 incidence and severity requires additional study.

## Data Availability Statement

The original contributions presented in the study are included in the article/Supplementary Material, further inquiries can be directed to the corresponding authors.

## Author Contributions

VC and PK conceived the idea for the review. VC, AK, MM, and PK designed and undertook the literature review. VC, HS, MM, AK, and RS screened the articles and extracted the data. VC and MM performed the statistical analysis, figures, and appendix and analyzed and interpreted the data. VC, HS, AK, MM, RS, DV, JM, and PK wrote the first draft of the manuscript. VC, AK, HS, DV, JM, and PK revised the subsequent drafts of the manuscript. All authors reviewed the final draft of the manuscript.

## Conflict of Interest

The authors declare that the research was conducted in the absence of any commercial or financial relationships that could be construed as a potential conflict of interest.

## Publisher’s Note

All claims expressed in this article are solely those of the authors and do not necessarily represent those of their affiliated organizations, or those of the publisher, the editors and the reviewers. Any product that may be evaluated in this article, or claim that may be made by its manufacturer, is not guaranteed or endorsed by the publisher.
