# Supplementary material for: Association of Lipid Levels With COVID-19 Infection, Disease Severity and Mortality: A Systematic Review and Meta-Analysis
Source: Front Cardiovasc Med. 2022 Mar 24;9:862999. doi: 10.3389/fcvm.2022.862999 (PMC8988060; doi:10.3389/fcvm.2022.862999)
Supplement: Supplementary file 1 [file Data_Sheet_1.PDF]

Supplementary Appendix

Section-I

Search Strategy

PubMED -162 studies

"COVID-19" [Supplementary Concept] OR "severe acute respiratory syndrome coronavirus 2" [Supplementary Concept] OR 2019 novel coronavirus [tw] OR SARS2 [tw] OR coronavirus disease-19 OR COVID-19 [tw] OR "COVID 19"[tw] OR "COVID19"[tw] OR "COVID2019"[tw] OR "COVID 2019"[tw] OR "COVID-2019"[tw] OR "novel coronavirus"[tw] OR "new coronavirus"[tw] OR "novel corona virus"[tw] OR "new corona virus"[tw] OR "SARS-CoV-2"[tw] OR "SARSCoV2"[tw] OR "SARS-CoV2"[tw] OR "2019nCoV"[tw] OR "2019-nCoV"[tw] OR "2019 coronavirus"[tw] OR "2019 corona virus"[tw] OR "coronavirus disease 2019"[tw] OR "severe acute respiratory syndrome coronavirus 2"[nm] OR "severe acute respiratory syndrome coronavirus 2"[tw] OR "sars-coronavirus-2"[tw] OR "coronavirus disease 2019"[tw] OR "corona virus disease 2019"[tw] OR (wuhan[tw] AND ("2019/01/01"[PDAT]: "3000/12/31"[PDAT]))

AND

"Cholesterol, LDL"[Mesh] OR "Lipoproteins, LDL"[Mesh] OR "Lipoproteins, HDL"[Mesh] OR "Cholesterol, HDL"[Mesh] OR "Triglycerides"[Mesh] OR Dyslipidemias[MeSH] OR Hyperlipidemias[MeSH] OR Hypertriglyceridemia[MeSH] OR Hypercholesterolemia[MeSH] OR LDL[tiab] OR HDL[tiab] OR Cholestrol[tiab] OR lipoprotein[tiab] OR triglyceride[tiab] OR Lipid profile[tiab] OR Lipemia[tiab] OR Lipidemia[tiab] OR Serum lipid*[tiab] OR Dyslipidemia*[tiab] OR Dyslipoproteinemia*[tiab] OR Hyperlipidemia*[tiab] OR Hyperlipemia*[tiab] OR Hypertriglyceridemia[tiab] OR Hypercholesterolemia[tiab] OR Elevated serum cholesterol[tiab] OR High Cholesterol Level*[tiab] OR Hypercholesteremia[tiab] OR "low density lipoprotein":[tiab] OR "high density lipoprotein"[tiab] OR cholesterol[tiab]

AND

("Observational Study"[Publication Type] OR "cohort"[tiab] OR "case control"[tiab] OR "case controlled"[tiab] OR (("retrospective"[tiab] OR "retrospectively"[tiab] OR "prospective"[tiab] OR "observational"[tiab] OR "longitudinal"[tiab] OR "longitudinally"[tiab] OR "follow up"[tiab]) AND ("study"[tiab] OR "studies"[tiab])) OR "clinical study"[tiab] OR "clinical studies"[tiab] OR "validation study"[tiab] OR ("study"[tiab] AND "participants"[tiab])) NOT ("animals"[mh] NOT ("animals"[mh] AND "humans"[mh]))

EMBASE -165 studies

'2019 novel coronavirus'/exp OR SARS2:ab,ti,kw OR ‘Wuhan coronavirus’:ab,ti,kw OR ‘Wuhan seafood market pneumonia virus’:ab,ti,kw OR coronavirus disease-19:ab,ti,kw OR COVID-19:ab,ti,kw OR ‘COVID 19’:ab,ti,kw OR ‘COVID19’:ab,ti,kw OR ‘COVID2019’:ab,ti,kw OR ‘COVID 2019’:ab,ti,kw OR ‘COVID-2019’:ab,ti,kw OR ‘novel coronavirus’:ab,ti,kw OR ‘new coronavirus’:ab,ti,kw OR ‘novel corona virus’:ab,ti,kw OR ‘new corona virus’:ab,ti,kw OR ‘SARS-CoV-2 ’:ab,ti,kw OR ‘SARSCoV2’:ab,ti,kw OR ‘SARS-CoV2:ab,ti,kw’ OR ‘2019nCo:ab,ti,kw V’:ab,ti,kw OR ‘2019-nCoV’:ab,ti,kw OR ‘2019 coronavirus’:ab,ti,kw OR ‘2019 corona virus’:ab,ti,kw OR ‘coronavirus disease 2019’:ab,ti,kw OR ‘severe acute respiratory syndrome coronavirus 2’:ab,ti,kw OR ‘severe acute respiratory syndrome coronavirus 2’:ab,ti,kw OR ‘sars-coronavirus-2’:ab,ti,kw OR ‘coronavirus disease 2019’:ab,ti,kw OR ‘corona virus disease 2019’:ab,ti,kw OR (wuhan:ti,ab,kw AND [2019-2020]/py)

AND

'low density lipoprotein cholesterol'/exp OR 'high density lipoprotein cholesterol'/exp OR 'triacylglycerol'/exp OR ‘low density lipoprotein’:ab,ti,kw OR ‘high density lipoprotein’:ab,ti,kw OR ‘triacylglycerol’:ab,ti,kw OR ‘LDL’:ab,ti,kw OR ‘HDL’:ab,ti,kw OR ‘Lipid profile’:ab,ti,kw OR ‘cholesterol’:ab,ti,kw

AND

('case control study'/exp OR 'prospective study'/exp OR 'retrospective study'/exp OR 'cross-sectional study'/exp OR 'cohort analysis'/exp OR 'observational study'/exp OR 'cohort':ti,ab OR 'case control':ti,ab OR 'retrospective':ti,ab OR 'retrospectively':ti,ab OR 'prospective':ti,ab OR 'observational':ti,ab OR 'longitudinal':ti,ab OR 'longitudinally':ti,ab) NOT ('animal'/exp NOT ('animal'/exp AND 'human'/exp))

Section-II

Supplementary Table 1: Characteristics of Included Studies

Name of the Author	Month and year of publication	Country of study	Number of centers	Study design	Study duration	COVID vs Healthy adults	Severe vs non-severe COVID-19	Alive vs dead	Total cholesterol	LDL	HDL	Triglycerides
Huang et al.(1)	Feb 2021	China	Single Center	Retrospective Cohort	Before Jun 2020	*	*		*	*	*	*
Li et al.(2)	Jun 2021	China	Single Center	Retrospective Cohort	Jan 2020 - Apr 2020			*	*	*	*	
Ding et al.(3)	Nov 2020	China	Single Center	Retrospective Cohort	Jan 2020 - Mar 2020				*	*	*	*
Li et al. (4)	Mar 2021	China	Single Center	Retrospective Cohort	Feb 2020 - Aug 2020		*	*	*	*	*	*
Zhou et al. (5)	Apr 2021	China	Multi-center	Retrospective Cohort	Jan 2020 - Aug 2020		*		*	*	*	*
Wei et al.(6)	May 2020	China	Single Center	Retrospective Cohort	Feb 2020 - Mar 2020		*		*	*	*	*
Yue et al.(7)	Jun 2021	China	Single Center	Retrospective Cohort	Feb 2020 - Mar 2020			*		*	*	*
Hu et al.(8)	Jul 2020	China	Single Center	Retrospective Cohort	Jan 2020 - Feb 2020	*	*		*	*	*	*
Wang et al.(9)	Jul 2020	China	Single Center	Retrospective Cohort	Jan 2020 - Feb 2020		*		*			*
Li et al.(2)	Feb 2021	China	Single Center	Retrospective Cohort	Jan 2020 - Apr 2020		*		*	*		
Wang et al.(10)	Sep 2020	China	Single Center	Retrospective Cohort	Jan 2020 - Mar 2020		*	*		*	*	
Qin et al.(11)	Sep 2020	China	Multi-center	Retrospective Cohort	Feb 2020 - Apr 2020		*		*	*	*	*
Zhao et al.(12)	May 2021	China	Single Center	Retrospective Cohort	Jan 2020 - Feb 2020		*			*		
Ouyang et al.(13)	Dec 2020	China	Single Center	Retrospective Cohort	Jan 2020 - Mar 2020				*	*	*	*
Zhihua Lv et	Nov 2020	China	Single Center	Retrospective Cohort	Feb 2020 - Feb 2020	*	*		*	*	*	*

al.(14)												
Sun et al.(15)	Dec 2020	China	Single Center	Retrospective Cohort	Feb 2020 - Apr 2020		*	*	*	*	*	
Zhong et al.(16)	May 2021	China	Multi-center	Retrospective Cohort	Feb 2020 - Feb 2020		*		*			
Wang et al.(17)	Aug 2020	France	Single Center	Retrospective Cohort	Mar 2020 - Apr 2020	*	*		*	*	*	*
Tanaka et al.(18)	Sep 2020	France	Single Center	Prospective Cohort	Mar 2020 - Apr 2020			*	*	*	*	*
Begue et al.(19)	Jan 2021	France	Single Center	Prospective Cohort	Before Sept 2020	*				*	*	*
Salari et al.(20)	Dec 2020	Iran	Single Center	Retrospective Cohort	Apr 2020 - Aug 2020			*	*	*	*	*
Bellia et al.(21)	May 2021	Italy	Single Center	Retrospective Cohort	Mar 2020 - May 2020		*		*	*	*	*
Alcantara-Alonso et al.(22)	Apr 2021	Mexico	Single Center	Retrospective Cohort	May 2020 - Oct 2020		*		*	*	*	*
Masana et al.(23)	Mar 2021	Spain	Multi-center	Retrospective Cohort	Till May 2020		*		*	*	*	*
Aparisi et al.(24)	Jul 2021	Spain	Single Center	Retrospective Cohort	Mar 2020 - May 2020			*	*	*	*	
Yildirim et al.(25)	Jan 2021	Turkey	Single Center	Retrospective Cohort	Mar 2020 - May 2020			*	*	*	*	
Hilser et al.(26)	Jan 2021	UK	Multi-center	Retrospective Cohort	2006	*				*	*	*
Ho et al.(27)	Oct 2020	UK	Multi-center	Retrospective Cohort	Mar 2020 -May 2020	*					*	
Henry et al.(28)	Apr 2021	USA	Single Center	Prospective Cohort	Before March 2021		*		*	*	*	*

Supplementary Table 2: New Castle Ottawa Quality assessment scale for the included studies

Name of the Author	New Castle-Ottawa Quality assessment Scale (Total score=9)								Total
	Representativeness of exposed	Selection of the non-exposed	Ascertainment of exposure	Outcome of interest was not present at the start	Comparability	Assessment of outcome	Long enough follow up	Adequacy of follow up	
Huang et al.(1)	*	*	*	*	*	*	*	*	8
Li et al.(2)	*	*	*	*	*	*	*	*	8
Ding et al.(3)	*	*	*	*	*	*	*	*	8
Li et al. (4)	*	*	*	*	*	*	*	*	8
Zhou et al. (5)	*	*	*	*	*	*	*	*	8
Wei et al.(6)	*			*		*	*	*	5
Yue et al.(7)	*	*	*	*	*	*	*	*	8
Hu et al.(8)	*	*	*	*	*	*	*		7
Wang et al.(9)	*	*	*	*	*	*	*		7
Li et al.(2)	*	*	*	*	*	*	*	*	8
Wang et al.(10)	*	*	*	*	*	*	*		7
Qin et al.(11)	*	*	*	*	*	*	*	*	8
Zhao et al.(12)	*	*	*	*	*	*	*	*	8
Ouyang et al.(13)	*	*	*	*	*	*	*	*	8

Zhihua Lv et al.(14)	*	*	*	*	**	*	*	*	9
Sun et al.(15)			*	*	*	*	*	*	5
Zhong et al.(16)	*		*	*	*	*	*	*	7
Wang et al.(17)	*	*	*	*	*	*	*		7
Tanaka et al.(18)	*		*	*	*	*	*		6
Begue et al.(19)	*	*	*	*	*	*	*	*	8
Salari et al.(20)	*			*	*	*			4
Bellia et al.(21)	*	*	*	*	*	*	*	*	8
Alcantara-Alonso et al.(22)	*	*	*	*	*	*	*	*	8
Masana et al.(23)	*	*	*	*	*	*	*	*	8
Aparisi et al.(24)	*	*	*	*	*	*	*	*	8
Yildirim et al.(25)	*		*	*	*	*	*	*	7
Hilser et al.(26)	*	*	*	*	*	*	*	*	8
Ho et al.(27)	*	*	*	*	*	*	*	*	8
Henry et al.(28)	*	*	*	*	*				5

Section-III

Supplementary Figures

Supplementary Figure 1: Bubble plot showing meta-regression of mean difference of Lipid levels (mg/dL) between severe and non-severe COVID-19 with mean difference in age (years) between severe and non-severe COVID-19.

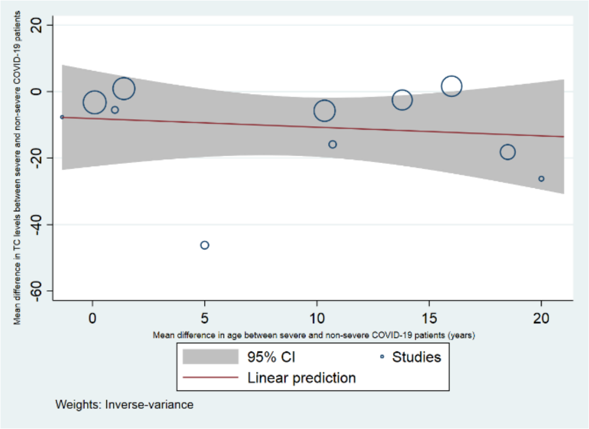

A)

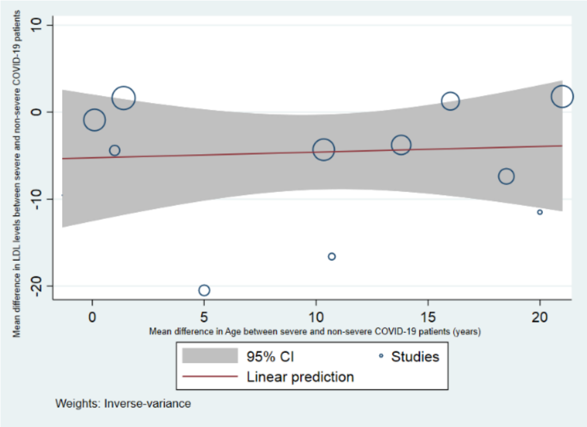

B)

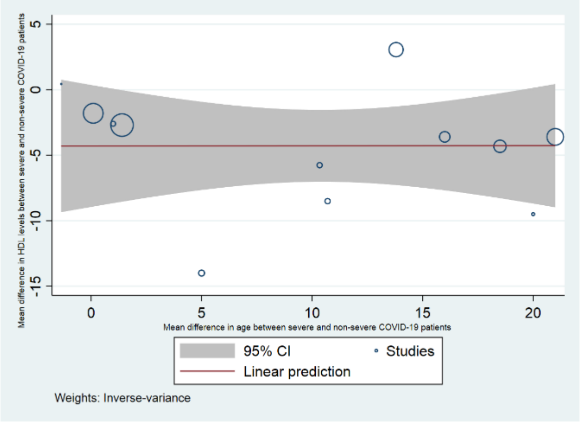

C)

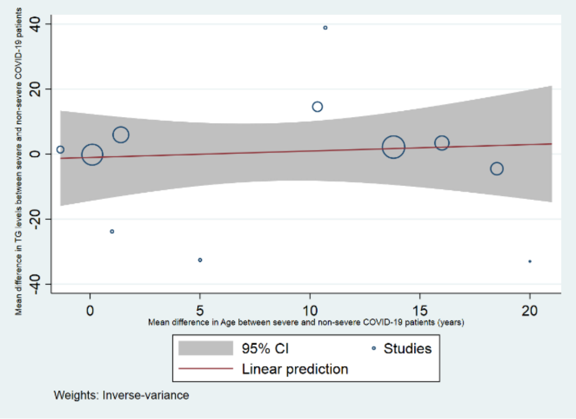

D)

Supplementary Figure 2: Bubble plot showing meta-regression of mean difference of Lipid levels (mg/dL) between severe and non-severe COVID-19 with difference in proportion of male sex (%) between severe and non-severe COVID-19.

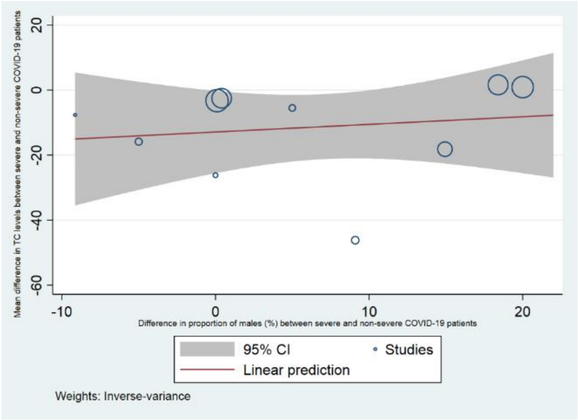

A)

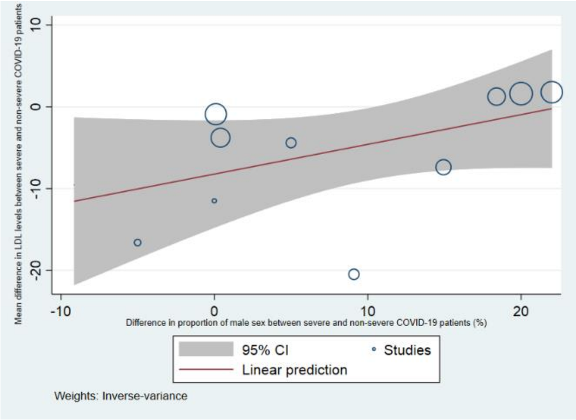

B)

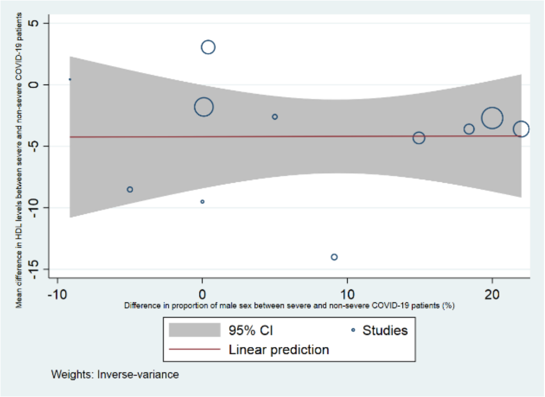

C)

Supplementary Figure 3: Bubble plot showing meta-regression of mean difference of Lipid levels (mg/dL) between severe and non-severe COVID-19 with difference in proportion of diabetes (%) between severe and non-severe COVID-19.

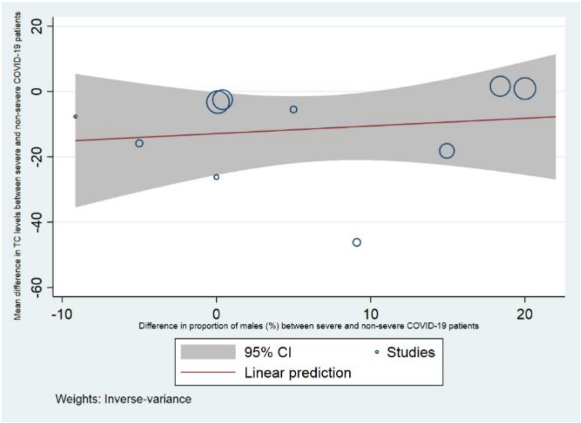

A)

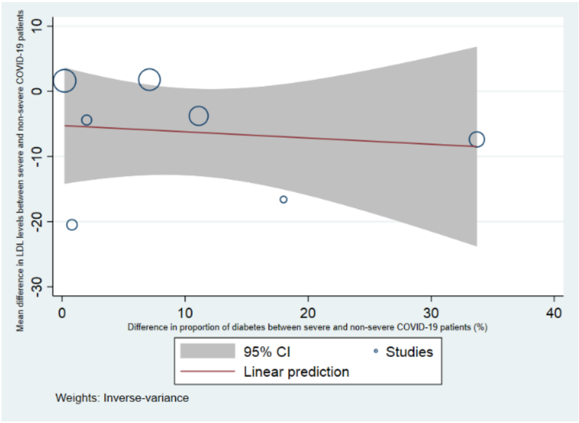

B)

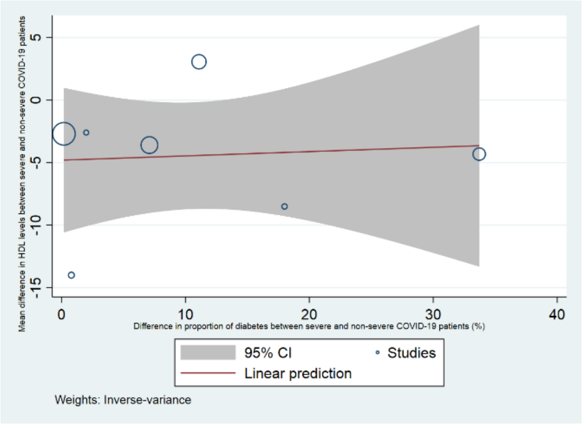

C)

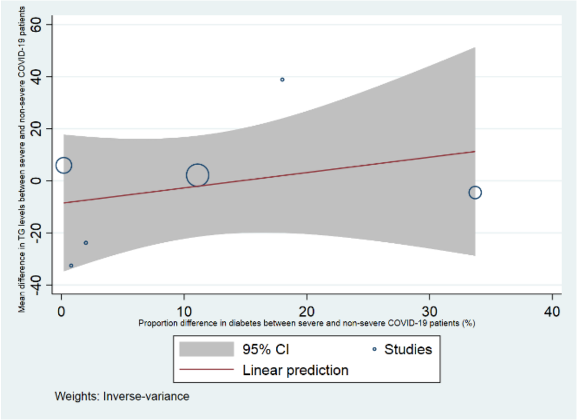

D)

Supplementary Figure 4: Bubble plot showing meta-regression of mean difference of Lipid levels (mg/dL) between severe and non-severe COVID-19 with difference in proportion of hypertension (%) between severe and non-severe COVID-19.

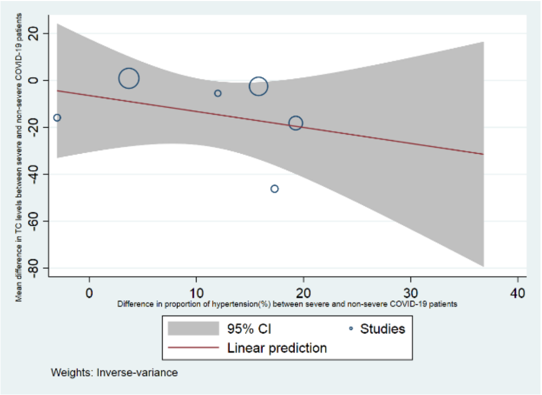

A)

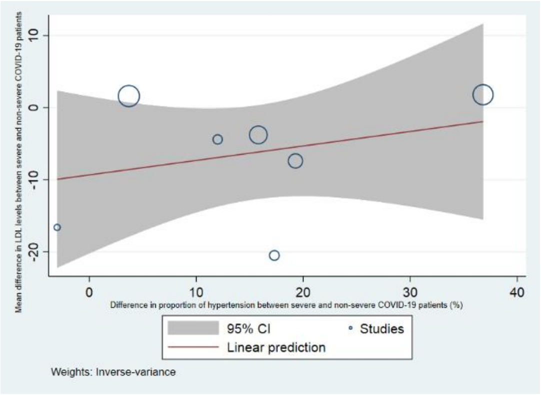

B)

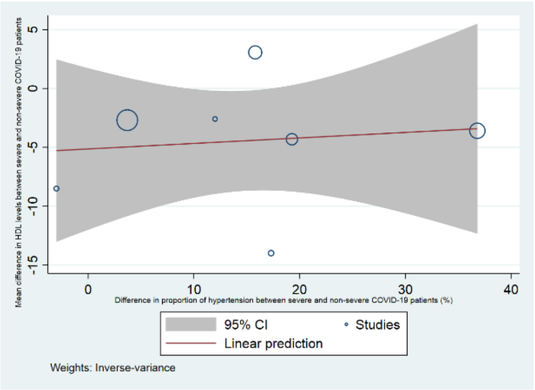

C)

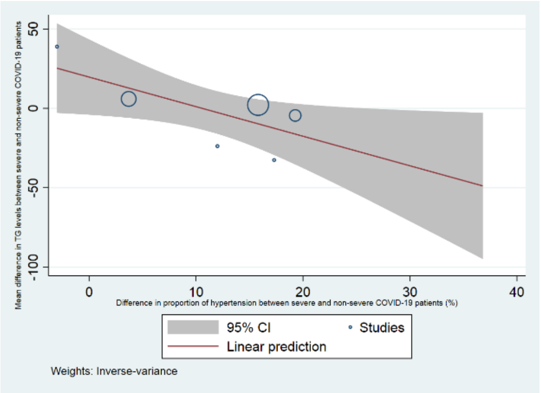

D)

Supplementary Figure 5: Bubble plot showing meta-regression of mean difference of Lipid levels (mg/dL) between severe and non-severe COVID-19 with difference in proportion of CAD (%) between severe and non-severe COVID-19.

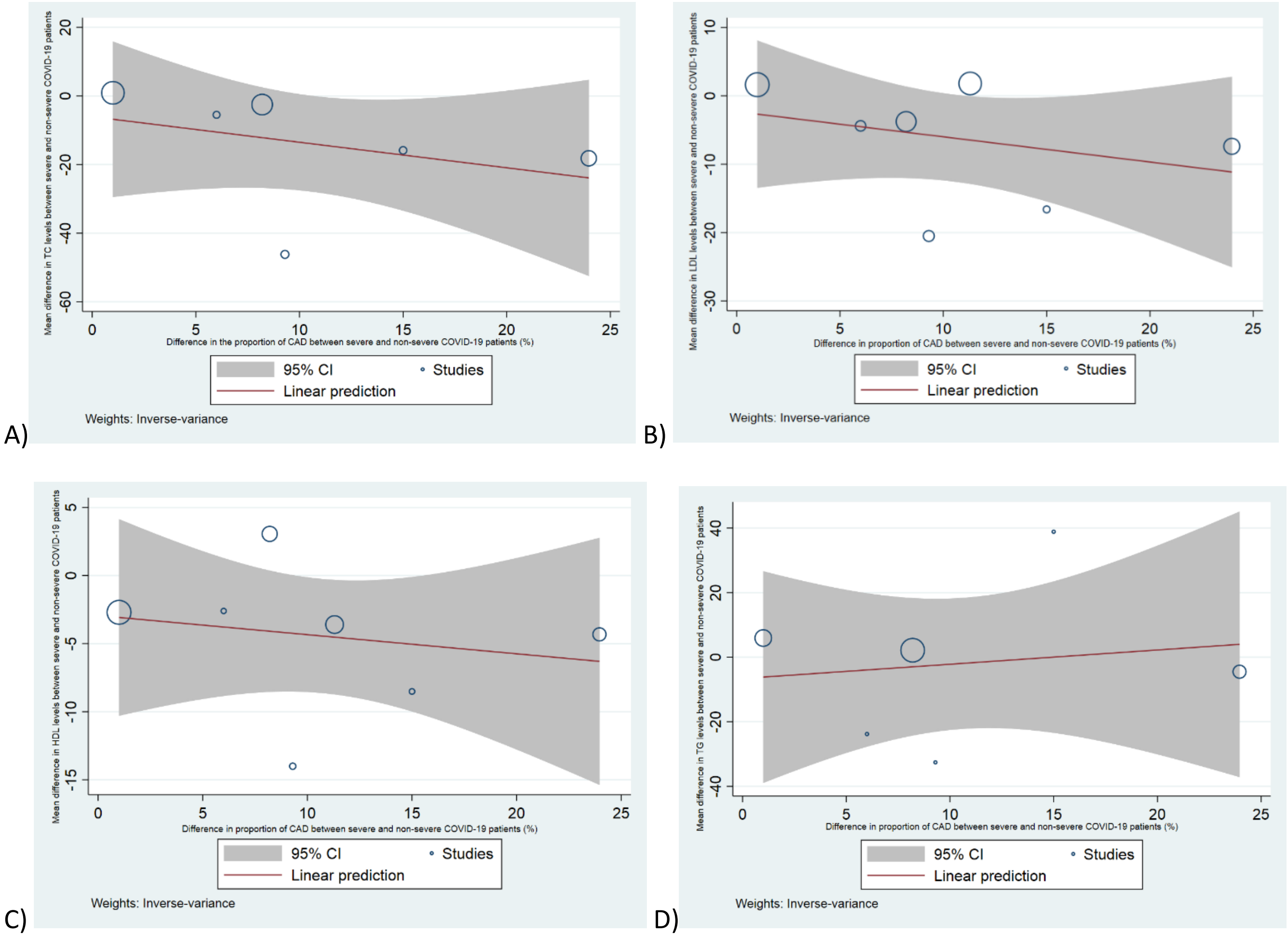

Section-IV

Section and Topic	Item #	Checklist item	Location where item is reported
**TITLE**			
Title	1	Identify the report as a systematic review.	Pg.1
**ABSTRACT**			
Abstract	2	See the PRISMA 2020 for Abstracts checklist.	Pg.2
**INTRODUCTION**			
Rationale	3	Describe the rationale for the review in the context of existing knowledge.	Pg.4
Objectives	4	Provide an explicit statement of the objective(s) or question(s) the review addresses.	Pg.4
**METHODS**			
Eligibility criteria	5	Specify the inclusion and exclusion criteria for the review and how studies were grouped for the syntheses.	Pg.4
Information sources	6	Specify all databases, registers, websites, organisations, reference lists and other sources searched or consulted to identify studies. Specify the date when each source was last searched or consulted.	Pg.4,5
Search strategy	7	Present the full search strategies for all databases, registers and websites, including any filters and limits used.	Supplementary document – Section 1
Selection process	8	Specify the methods used to decide whether a study met the inclusion criteria of the review, including how many reviewers screened each record and each report retrieved, whether they worked independently, and if applicable, details of automation tools used in the process.	Pg.4,5
Data collection process	9	Specify the methods used to collect data from reports, including how many reviewers collected data from each report, whether they worked independently, any processes for obtaining or confirming data from study investigators, and if applicable, details of automation tools used in the process.	Pg.4,5
Data items	10a	List and define all outcomes for which data were sought. Specify whether all results that were compatible with each outcome domain in each study were sought (e.g. for all measures, time points, analyses), and if not, the methods used to decide which results to collect.	Pg.5,6
	10b	List and define all other variables for which data were sought (e.g. participant and intervention characteristics, funding sources). Describe any assumptions made about any missing or unclear information.	Pg.5,6
Study risk of bias assessment	11	Specify the methods used to assess risk of bias in the included studies, including details of the tool(s) used, how many reviewers assessed each study and whether they worked independently, and if applicable, details of automation tools used in the process.	Pg.5
Effect measures	12	Specify for each outcome the effect measure(s) (e.g. risk ratio, mean difference) used in the synthesis or presentation of results.	Pg.5,6
Synthesis methods	13a	Describe the processes used to decide which studies were eligible for each synthesis (e.g. tabulating the study intervention characteristics and comparing against the planned groups for each synthesis (item #5)).	Pg.6
	13b	Describe any methods required to prepare the data for presentation or synthesis, such as handling of missing summary statistics, or data conversions.	Pg.6
	13c	Describe any methods used to tabulate or visually display results of individual studies and syntheses.	Pg.5,6
	13d	Describe any methods used to synthesize results and provide a rationale for the choice(s). If meta-analysis was performed, describe the model(s), method(s) to identify the presence and extent of statistical heterogeneity, and software package(s) used.	Pg.5,6
	13e	Describe any methods used to explore possible causes of heterogeneity among study results (e.g. subgroup analysis, meta-regression).	Pg.6
	13f	Describe any sensitivity analyses conducted to assess robustness of the synthesized results.	-
Reporting bias assessment	14	Describe any methods used to assess risk of bias due to missing results in a synthesis (arising from reporting biases).	Supplementary table 2
Certainty assessment	15	Describe any methods used to assess certainty (or confidence) in the body of evidence for an outcome.	Pg.6

Section and Topic	Item #	Checklist item	Location where item is reported
**RESULTS**			
Study selection	16a	Describe the results of the search and selection process, from the number of records identified in the search to the number of studies included in the review, ideally using a flow diagram.	Pg.7
	16b	Cite studies that might appear to meet the inclusion criteria, but which were excluded, and explain why they were excluded.	Pg.7
Study characteristics	17	Cite each included study and present its characteristics.	Supplementary Table 1
Risk of bias in studies	18	Present assessments of risk of bias for each included study.	Supplementary Table 2
Results of individual studies	19	For all outcomes, present, for each study: (a) summary statistics for each group (where appropriate) and (b) an effect estimate and its precision (e.g. confidence/credible interval), ideally using structured tables or plots.	Figure 2
Results of syntheses	20a	For each synthesis, briefly summarise the characteristics and risk of bias among contributing studies.	Pg.6,7,8
	20b	Present results of all statistical syntheses conducted. If meta-analysis was done, present for each the summary estimate and its precision (e.g. confidence/credible interval) and measures of statistical heterogeneity. If comparing groups, describe the direction of the effect.	Pg.6,7,8
	20c	Present results of all investigations of possible causes of heterogeneity among study results.	Pg.8
	20d	Present results of all sensitivity analyses conducted to assess the robustness of the synthesized results.	-
Reporting biases	21	Present assessments of risk of bias due to missing results (arising from reporting biases) for each synthesis assessed.	Supplementary Table 2
Certainty of evidence	22	Present assessments of certainty (or confidence) in the body of evidence for each outcome assessed.	Pg.6,7,8
**DISCUSSION**			
Discussion	23a	Provide a general interpretation of the results in the context of other evidence.	Pg.8
	23b	Discuss any limitations of the evidence included in the review.	Pg.10
	23c	Discuss any limitations of the review processes used.	Pg.10
	23d	Discuss implications of the results for practice, policy, and future research.	Pg.10
**OTHER INFORMATION**			
Registration and protocol	24a	Provide registration information for the review, including register name and registration number, or state that the review was not registered.	Not registered
	24b	Indicate where the review protocol can be accessed, or state that a protocol was not prepared.	Attached document
	24c	Describe and explain any amendments to information provided at registration or in the protocol.	None
Support	25	Describe sources of financial or non-financial support for the review, and the role of the funders or sponsors in the review.	Pg.11
Competing interests	26	Declare any competing interests of review authors.	Pg.11
Availability of data, code and other materials	27	Report which of the following are publicly available and where they can be found: template data collection forms; data extracted from included studies; data used for all analyses; analytic code; any other materials used in the review.	Pg.11

Section-V

References in Supplementary Document

1. Huang S, Zhou C, Yuan Z, Xiao H, Wu X. The clinical value of high-density lipoprotein in the evaluation of new coronavirus pneumonia. Adv. Clin. Exp. Med. 2021;30:153–156.

2. Li Y, Zhang Y, Lu R, et al. Lipid metabolism changes in patients with severe COVID-19. Clin. Chim. Acta 2021;517:66–73.

3. Ding X, Zhang J, Liu L, et al. High-density lipoprotein cholesterol as a factor affecting virus clearance in covid-19 patients. Respir. Med. 2020;175:106218. Available at: https://doi.org/10.1016/j.rmed.2020.106218.

4. Li G, Du L, Cao X, et al. Follow-up study on serum cholesterol profiles and potential sequelae in recovered COVID-19 patients. BMC Infect. Dis. 2021;21:1–10.

5. Zhou J, Lee S, Wang X, et al. Development of a multivariable prediction model for severe COVID-19 disease: a population-based study from Hong Kong. npj Digit. Med. 2021 41 2021;4:1–9. Available at: https://www.nature.com/articles/s41746-021-00433-4. Accessed November 14, 2021.

6. Wei X, Zeng W, Su J, et al. Hypolipidemia is associated with the severity of COVID-19. J. Clin. Lipidol. 2020;14:297–304.

7. Yue J, Xu H, Zhou Y, et al. Dyslipidemia Is Related to Mortality in Critical Patients With Coronavirus Disease 2019: A Retrospective Study. Front. Endocrinol. (Lausanne). 2021;12.

8. Hu X, Chen D, Wu L, He G, Ye W. Declined serum high density lipoprotein cholesterol is associated with the severity of COVID-19 infection. Clin. Chim. Acta 2020;510:105–110. Available at: https://doi.org/10.1016/j.cca.2020.07.015.

9. Wang D, Li R, Wang J, et al. Correlation analysis between disease severity and clinical and biochemical characteristics of 143 cases of COVID-19 in Wuhan, China: A descriptive study. BMC Infect. Dis. 2020;20:1–9.

10. Wang G, Zhang Q, Zhao X, et al. Low high-density lipoprotein level is correlated with the severity of COVID-19 patients: An observational study. Lipids Health Dis. 2020;19:1–7.

11. Qin C, Minghan H, Ziwen Z, Yukun L. Alteration of lipid profile and value of lipids in the prediction of the length of hospital stay in COVID-19 pneumonia patients. Food Sci. Nutr. 2020;8:6144–6152.

12. Zhao M, Luo Z, He H, et al. Decreased Low-Density Lipoprotein Cholesterol Level Indicates Poor Prognosis of Severe and Critical COVID-19 Patients: A Retrospective, Single-Center Study. Front. Med. 2021;8:1–15.

13. Ouyang SM, Zhu HQ, Xie YN, et al. Temporal changes in laboratory markers of survivors and non-survivors of adult inpatients with COVID-19. BMC Infect. Dis. 2020;20:1–10.

14. Lv Z, Wang W, Qiao B, et al. The prognostic value of general laboratory testing in patients with COVID-19. J. Clin. Lab. Anal. 2021;35:e23668. Available at: https://onlinelibrary.wiley.com/doi/full/10.1002/jcla.23668. Accessed November 14, 2021.

15. Sun JT, Chen Z, Nie P, et al. Lipid Profile Features and Their Associations With Disease Severity and Mortality in Patients With COVID-19. Front. Cardiovasc. Med. 2020;0:290.

16. Zhong P, Wang Z, Du Z. Serum triglyceride levels and related factors as prognostic indicators in COVID-19 patients: A retrospective study. Immunity, Inflamm. Dis. 2021;9:1055–1060.

17. Ressaire Q, Dudoignon E, Moreno N, Coutrot M, Dépret F. Low total cholesterol blood level is correlated with pulmonary severity in COVID-19 critical ill patients. Anaesthesia, Crit. care pain Med. 2020;39:733–735. Available at: https://pubmed.ncbi.nlm.nih.gov/32866665/. Accessed November 18, 2021.

18. Tanaka S, De Tymowski C, Assadi M, et al. Lipoprotein concentrations over time in the intensive care unit COVID-19 patients: Results from the ApoCOVID study. PLoS One 2020;15:1–15.

19. Begue F, Tanaka S, Mouktadi Z, et al. Altered high-density lipoprotein composition and functions during severe COVID-19. Sci. Rep. 2021;11:2291. Available at: https://doi.org/10.1038/s41598-021-81638-1. Accessed May 15, 2021.

20. Salari A, Mahdavi-Roshan M, Ghorbani Z, et al. An investigation of risk factors of in-hospital death due to COVID-19: a case-control study in Rasht, Iran. Ir. J. Med. Sci. 2021;190:1321–1333. Available at: https://pubmed.ncbi.nlm.nih.gov/33449333/. Accessed November 18, 2021.

21. Bellia A, Andreadi A, Giudice L, et al. Atherogenic dyslipidemia on admission is associated with poorer outcome in people with and without diabetes hospitalized for covid-19. Diabetes Care 2021;44:2149–2157.

22. Alcántara-Alonso E, Molinar-Ramos F, González-López JA, et al. High triglyceride to HDL-cholesterol ratio as a biochemical marker of severe outcomes in COVID-19 patients. Clin. Nutr. ESPEN 2021;44:437–444. Available at: https://pubmed.ncbi.nlm.nih.gov/34330502/. Accessed November 18, 2021.

23. Masana L, Correig E, Ibarretxe D, et al. Low HDL and high triglycerides predict COVID-19 severity. Sci. Rep. 2021;11:7217. Available at: www.nature.com/scientificreports. Accessed May 15, 2021.

24. Aparisi Á, Iglesias-Echeverría C, Ybarra-Falcón C, et al. Low-density lipoprotein cholesterol levels are associated with poor clinical outcomes in COVID-19. Nutr. Metab. Cardiovasc. Dis. 2021;31:2619–2627. Available at: https://pubmed.ncbi.nlm.nih.gov/34353699/. Accessed November 18, 2021.

25. Turgay Yıldırım Ö, Kaya Ş. The atherogenic index of plasma as a predictor of mortality in patients with COVID-19. Hear. Lung 2021;50:329–333.

26. Hilser JR, Han Y, Biswas S, et al. Association of serum HDL-cholesterol and apolipoprotein A1 levels with risk of severe SARS-CoV-2 infection. J. Lipid Res. 2021;62. Available at: https://pubmed.ncbi.nlm.nih.gov/33667465/. Accessed November 18, 2021.

27. Ho FK, Celis-Morales CA, Gray SR, et al. Modifiable and non-modifiable risk factors for COVID-19, and comparison to risk factors for influenza and pneumonia: Results from a UK Biobank prospective cohort study. BMJ Open 2020;10.

28. Henry BM, Szergyuk I, de Oliveira MHS, et al. Alterations in the lipid profile associate with a dysregulated inflammatory, prothrombotic, anti-fibrinolytic state and development of severe acute kidney injury in coronavirus disease 2019 (COVID-19): A study from Cincinnati, USA. Diabetes Metab. Syndr. 2021;15:863–868. Available at: https://pubmed.ncbi.nlm.nih.gov/33878674/. Accessed November 18, 2021.
