# Supplementary material for: Association of Lipid Levels With COVID-19 Infection, Disease Severity and Mortality: A Systematic Review and Meta-Analysis
Source: Front Cardiovasc Med. 2022 Mar 24;9:862999. doi: 10.3389/fcvm.2022.862999 (PMC8988060; doi:10.3389/fcvm.2022.862999)
Supplement: Supplementary file 2 [file Data_Sheet_2.PDF]

Study Protocol

Association of lipid levels with COVID-19 infection, disease severity and mortality: A systematic review and meta-analysis

Background

Coronavirus disease 2019 (COVID-19), caused by SARS-CoV-2, is currently the leading cause of death due to a single infectious agent[1]. Several investigators have reported an association between COVID-19 severity and low serum levels of high-density lipoprotein cholesterol (HDL)[3,4], but the magnitude of the association between COVID-19 and total cholesterol (TC), low density lipoprotein cholesterol (LDL), and triglycerides (TG) is inconsistent. This finding is in agreement with similar observations in patients with other infections, such as HIV, dengue, and tuberculosis[5,6].

Serum lipid levels may serve as biomarkers predicting disease severity and mortality and provide insight into the pathogenesis of COVID-19. We plan to perform a systematic review of the literature and meta-analysis to determine differences in serum lipid levels, such as TC, LDL, HDL, and TG, between patients with COVID-19 and healthy controls, and to assess the association of these lipid levels with severity and mortality among patients with COVID-19.

Research question

To compare the serum levels of TC, LDL, HDL, and triglycerides (TG) between 1) patients with COVID-19 vs. healthy controls; 2) patients with severe COVID-19 vs. non-severe disease; 3) patients with COVID-19 who died vs. those who survived.

Electronic searches

We plan to include peer-reviewed English language articles from PubMed and Embase.

Study Selection

We plan to include Observational studies, such as prospective and retrospective cohort studies, and case-control studies. Cross-sectional studies, case reports, and case series will be excluded from this review. We will include studies that report serum levels of lipids, such as TC, LDL, HDL, and TG,

and at least one of the following: 1) difference in the lipid levels of patients with and without COVID-19; or 2) direct comparison of lipid levels at admission among COVID-19 patients with and without severe disease or 3) direct comparison of lipid levels at admission among patients with COVID-19 who died and those who survived.

Search Strategy

PubMED

"COVID-19" [Supplementary Concept] OR "severe acute respiratory syndrome coronavirus 2" [Supplementary Concept] OR 2019 novel coronavirus [tw] OR SARS2 [tw] OR coronavirus disease-19 OR COVID-19 [tw] OR "COVID 19"[tw] OR "COVID19"[tw] OR "COVID2019"[tw] OR "COVID 2019"[tw] OR "COVID-2019"[tw] OR "novel coronavirus"[tw] OR "new coronavirus"[tw] OR "novel corona virus"[tw] OR "new corona virus"[tw] OR "SARS-CoV-2"[tw] OR "SARSCoV2"[tw] OR "SARS-CoV2"[tw] OR "2019nCoV"[tw] OR "2019-nCoV"[tw] OR "2019 coronavirus"[tw] OR "2019 corona virus"[tw] OR "coronavirus disease 2019"[tw] OR "severe acute respiratory syndrome coronavirus 2"[nm] OR "severe acute respiratory syndrome coronavirus 2"[tw] OR "sars-coronavirus-2"[tw] OR "coronavirus disease 2019"[tw] OR "corona virus disease 2019"[tw] OR (wuhan[tw] AND (“2019/01/01”[PDAT]: “3000/12/31”[PDAT]))

AND

"Cholesterol, LDL"[Mesh] OR "Lipoproteins, LDL"[Mesh] OR "Lipoproteins, HDL"[Mesh] OR "Cholesterol, HDL"[Mesh] OR "Triglycerides"[Mesh] OR Dyslipidemias[MeSH] OR Hyperlipidemias[MeSH] OR Hypertriglyceridemia[MeSH] OR Hypercholesterolemia[MeSH] OR LDL[tiab] OR HDL[tiab] OR Cholestrol[tiab] OR lipoprotein[tiab] OR triglyceride[tiab] OR Lipid profile[tiab] OR Lipemia[tiab] OR Lipidemia[tiab] OR Serum lipid*[tiab] OR Dyslipidemia*[tiab] OR Dyslipoproteinemia*[tiab] OR Hyperlipidemia*[tiab] OR Hyperlipemia*[tiab] OR Hypertriglyceridemia[tiab] OR Hypercholesterolemia[tiab] OR Elevated serum cholesterol[tiab] OR High Cholesterol Level*[tiab] OR Hypercholesteremia[tiab] OR “low density lipoprotein”:[tiab] OR “high density lipoprotein”[tiab] OR cholesterol[tiab]

AND

("Observational Study"[Publication Type] OR "cohort"[tiab] OR "case control"[tiab] OR "case controlled"[tiab] OR (("retrospective"[tiab] OR "retrospectively"[tiab] OR "prospective"[tiab] OR "observational"[tiab] OR "longitudinal"[tiab] OR "longitudinally"[tiab] OR "follow up"[tiab]) AND ("study"[tiab] OR "studies"[tiab])) OR "clinical study"[tiab] OR "clinical studies"[tiab] OR "validation study"[tiab] OR ("study"[tiab] AND "participants"[tiab])) NOT ("animals"[mh] NOT ("animals"[mh] AND "humans"[mh]))

EMBASE

'2019 novel coronavirus'/exp OR SARS2:ab,ti,kw OR ‘Wuhan coronavirus’:ab,ti,kw OR ‘Wuhan seafood market pneumonia virus’:ab,ti,kw OR coronavirus disease-19:ab,ti,kw OR COVID-19:ab,ti,kw OR ‘COVID 19’:ab,ti,kw OR ‘COVID19’:ab,ti,kw OR ‘COVID2019’:ab,ti,kw OR ‘COVID 2019’:ab,ti,kw OR ‘COVID-2019’:ab,ti,kw OR ‘novel coronavirus’:ab,ti,kw OR ‘new coronavirus’:ab,ti,kw OR ‘novel corona virus’:ab,ti,kw OR ‘new corona virus’:ab,ti,kw OR ‘SARS-CoV-2 ’:ab,ti,kw OR ‘SARSCoV2’:ab,ti,kw OR ‘SARS-CoV2:ab,ti,kw’ OR ‘2019nCo:ab,ti,kw V’:ab,ti,kw OR ‘2019-nCoV’:ab,ti,kw OR ‘2019 coronavirus’:ab,ti,kw OR ‘2019 corona virus’:ab,ti,kw OR ‘coronavirus disease 2019’:ab,ti,kw OR ‘severe acute respiratory syndrome coronavirus 2’:ab,ti,kw OR ‘severe acute respiratory syndrome coronavirus 2’:ab,ti,kw OR ‘sars-coronavirus-2’:ab,ti,kw OR ‘coronavirus disease 2019’:ab,ti,kw OR ‘corona virus disease 2019’:ab,ti,kw OR (wuhan:ti,ab,kw AND [2019-2020]/py)

AND

'low density lipoprotein cholesterol'/exp OR 'high density lipoprotein cholesterol'/exp OR 'triacylglycerol'/exp OR ‘low density lipoprotein’:ab,ti,kw OR ‘high density lipoprotein’:ab,ti,kw OR ‘triacylglycerol’:ab,ti,kw OR ‘LDL’:ab,ti,kw OR ‘HDL’:ab,ti,kw OR ‘Lipid profile’:ab,ti,kw OR ‘cholesterol’:ab,ti,kw

AND

('case control study'/exp OR 'prospective study'/exp OR 'retrospective study'/exp OR 'cross-sectional study'/exp OR 'cohort analysis'/exp OR 'observational study'/exp OR 'cohort':ti,ab OR 'case control':ti,ab OR 'retrospective':ti,ab OR 'retrospectively':ti,ab OR 'prospective':ti,ab OR 'observational':ti,ab OR 'longitudinal':ti,ab OR 'longitudinally':ti,ab) NOT ('animal'/exp NOT ('animal'/exp AND 'human'/exp))

Other searches

We will also include the studies that are cited in other published journal articles and reviews. We will not use abstracts and conference reports if we are not able to obtain full information from the study authors. Studies in the pre-prints will not be included for this review because of the lack of peer review involved in the publication of these articles.

Methods

Type of studies included

Observational studies that reported a direct comparison of serum levels of TC, LDL, HDL, and triglycerides (TG) between 1) patients with COVID-19 vs. healthy controls; 2) patients with severe COVID-19 vs. non-severe disease; 3) patients with COVID-19 who died vs. those who survived, will be included in the review. Case reports, case series and other description studies will be excluded as measures of association would not be obtained from them.

Method of outcome measurement

Pooled mean differences (pMD) in lipid levels (mg/dL) for the above-mentioned groups will be obtained using random effects meta-analysis and assessed publication bias using funnel plots.

Mortality among SARS CoV-2 infection:

Defined as death as measured in studies following confirmed Coronavirus infection.

Severe disease among SARS CoV-2 infection:

Severe disease as defined by the American Thoracic Society guidelines for the treatment of Community-acquired Pneumonia or the Chinese National Health Commission guidelines for the Treatment of Novel Coronavirus infection

Data extraction (selection and coding)

Selection of studies

Data will be collected on the study characteristics, source of funding, type of lipid assessed (TC, LDL, HDL, and TG), year of publication, number of centers in each study, and study design. The articles retrieved from different databases will be imported into the Covidence platform for title/ abstract screening and full text screening. Each study will be independently screened by two reviewers based on the title and abstract of the articles obtained from the literature search. Conflicts on screening will be resolved through a third reviewer. This will be followed by the full text screening of each article independently by two reviewers. Conflicts on full-text screening will be resolved through a third reviewer.

Data extraction and management

Data from each study, meeting our inclusion criteria will be extracted by two reviewers, who will gather information on study characteristics, source of funding, type of lipid assessed (TC, LDL, HDL, and TG), year of publication, number of centers in each study, and study design. Disagreements will be resolved by ta third independent reviewer.

Dealing with duplicate and companion publications

The duplicate studies/articles will be automatically identified in the Covidence platform and be removed.

Data from clinical trials registers and CSR

We do not anticipate obtaining any clinical trial data on COVID-19 relevant to our research question.

Risk of bias (quality) assessment

Assessment of risk bias in included studies

Two authors will independently assess the risk of bias for each of the studies included for the analysis using the Newcastle Ottawa scale. Any discrepancy that is present will be resolved through discussion and/or by a third reviewer.

Each of the study will be assessed for the following:

Selection

1) Representativeness of the exposed cohort
2) Selection of the non-exposed cohort
3) Ascertainment of exposure
4) Demonstration that outcome of interest was not present at start of study

Comparability

1) Comparability of cohorts on the basis of the design or analysis

Outcome

1) Assessment of outcome
2) Was follow-up long enough for outcomes to occur
3) Adequacy of follow up of cohorts

Data synthesis

We will obtain pooled mean differences for the groups of interest using random-effects meta-analysis. We will analyze separately the mean levels of TC, LDL, HDL, and TG for each of the above comparisons. Statistical heterogeneity across the studies will be evaluated by forest plots, I^2 and Tau^2 statistics. We will assess publication bias using funnel plot, and Egger's test. Meta regression analyses for the mean difference of lipid levels across comparison groups will be performed against the mean

difference in age or difference in proportions of the confounding parameters such as sex, diabetes, hypertension, and coronary artery disease across the groups. All analyses will be carried out using the meta package in Stata (StataCorp, version 16)[7].

Assessment of reporting biases

Funnel plots and Egger's test will be used to assess publication bias, small study effects and error in methodological design.

Sensitivity and subgroup analyses

Decisions will be made during the review process regarding sensitivity and subgroup analysis.

Funding sources/sponsors

This systematic review will be supported by the NIAID/NIH UH3 AI122309 and K24AI143447 grants to PCK.

Conflicts of interest

Nothing to declare

Language

English

Subject index terms

COVID-19; Coronavirus; SARS-CoV-2; 2019-nCoV; viral Coronavirus disease 2019; severe acute respiratory syndrome coronavirus 2; HDL-cholesterol; LDL-cholesterol; Meta-regression, COVID-19, severity, mortality

References

1. Ahmad FB, Cisewski JA, Miniño A, Anderson RN. Provisional Mortality Data — United States, 2020. *MMWR Morb Mortal Wkly Rep*. 2021;70(14):519-522. doi:10.15585/mmwr.mm7014e1

2. Chidambaram V, Tun NL, Haque WZ, et al. Factors associated with disease severity and mortality among patients with COVID-19: A systematic review and meta-analysis. *PLoS One*. 2020;15(11 November). doi:10.1371/journal.pone.0241541

3. Feingold KR. Lipid and Lipoprotein Levels in Patients with COVID-19 Infections. *Endotext*. Published online November 15, 2020. Accessed October 16, 2021. https://www.ncbi.nlm.nih.gov/books/NBK564657/

4. Sun JT, Chen Z, Nie P, et al. Lipid Profile Features and Their Associations With Disease Severity and Mortality in Patients With COVID-19. *Front Cardiovasc Med*. 2020;0:290. doi:10.3389/FCVM.2020.584987

5. Feingold KR. *Lipid and Lipoprotein Levels in Patients with COVID-19 Infections*. MDText.com, Inc.; 2000. Accessed April 25, 2021. http://www.ncbi.nlm.nih.gov/pubmed/33237691

6. Chidambaram V, Zhou L, Castillo JR, et al. Higher serum cholesterol levels are associated with reduced systemic inflammation and mortality during tuberculosis treatment independent of body mass index. *Front Cardiovasc Med*. 2021;8:583. doi:10.3389/FCVM.2021.696517

7. StataCorp. 2019. Stata Statistical Software: Release 16. College Station TSL. Stata | StataCorp LLC. https://www.stata.com/company/
